# Adjuvant chemotherapy does not provide survival benefits to elderly patients with stage II colon cancer

**DOI:** 10.1038/s41598-019-48197-y

**Published:** 2019-08-14

**Authors:** Kil-yong Lee, Ji Won Park, Ki-young Lee, Sangsik Cho, Yoon-Hye Kwon, Min Jung Kim, Seung-Bum Ryoo, Seung-Yong Jeong, Kyu Joo Park

**Affiliations:** 1 Department of Surgery, Seoul National University College of Medicine, Seoul, Korea; 2 Cancer Research Institute, Seoul National University College of Medicine, Seoul, Korea

**Keywords:** Surgical oncology, Colon cancer

## Abstract

To date, the effect of adjuvant chemotherapy after curative resection in patients with stage II colon cancer remains controversial. Still, little is known about the effects of adjuvant chemotherapy in patients with stage II colon cancer who are older than 70 years, as most studies did not focus on this population. This study aimed to investigate the oncologic outcomes of elderly patients with stage II colon cancer who underwent curative resection with or without postoperative adjuvant chemotherapy. We retrospectively reviewed medical records of patients older than 70 years who underwent curative resection of stage II primary colon cancer during 2002–2015. Patients were classified into surgery alone (SA) and adjuvant chemotherapy (AC) groups and propensity score-matched at a 1:1 ratio using a logistic regression. The end points were recurrence-free (RFS), cancer-specific (CSS) and overall survival (OS). Of the 623 patients who met the criteria, 145 were assigned to each arm after propensity score matching. The mean ages of the SA and AC groups were 74.3 and 74.0 years, respectively. A log-rank test revealed no significant inter-group differences in RFS (p = 0.202), CSS (p = 0.486) or OS (p = 0.299). In a Cox regression analysis, adjuvant chemotherapy was not found to be an independent factor affecting RFS (p = 0.206), CSS (p = 0.487) or OS (p = 0.301). Adjuvant chemotherapy does not appear to yield survival benefits in elderly patients with stage II colon cancer.

## Introduction

The number of elderly patients diagnosed with colon cancer continues to increase worldwide, in parallel with population aging^1^. However, no guideline for the management of colon cancer in this population has been established because elderly patients generally have been excluded from randomized control studies^2^. A recent review has highlighted the problems of a lack of evidence and under-representation of elderly patients in clinical trials on the specific effects of adjuvant chemotherapy in elderly patients because of strict age-based inclusion and exclusion criteria^3^. In one study of data from Medicare and the Texas Cancer Registry, Zhao and colleagues reported that guideline-concordant treatment, including adjuvant chemotherapy, was associated with better survival outcomes among elderly patients with stage II and stage III colon cancer^4^. However, elderly patients tend to have a poorer general condition, compared to their younger counterparts, and may therefore face an increased risk of morbidity and mortality associated with chemotherapy-related adverse effects^5–7^.

The effect of adjuvant chemotherapy after curative resection in stage II colon cancer patients remains controversial. Some studies reported that adjuvant chemotherapy confers survival benefits^8,9^, whereas other recent studies suggest a lack of association with improved survival gain^9–12^. However, as most previous studies did not focus on patients older than 70 years, little information is available about the potential benefits of adjuvant chemotherapy for stage II colon cancer in this population. Therefore, we aimed to investigate the oncologic outcomes, including recurrence-free (RFS), cancer-specific (CSS) and overall survival (OS), in elderly patients with stage II colon cancer who underwent curative resection with or without adjuvant chemotherapy. We hypothesize that these two groups of patients would achieve different survival outcomes.

## Materials and Methods

This study was approved by the institutional review board (IRB) of Seoul National University Hospital. The IRB waived the requirement for informed consent because of the retrospective nature of the study.

### Patients

We retrospectively reviewed the medical records of patients older than 70 years who underwent curative resection of stage II primary colon cancer at Seoul National University Hospital from 2002 to 2015. Patients with a history of other malignancy or missing data regarding the body mass index (BMI), American Society of Anesthesiologists (ASA) classification and/or pathologic results (e.g., perineural, venous, lymphatic invasion) were excluded. The remaining patients were divided into two groups: the adjuvant chemotherapy (AC) group comprised patients who received postoperative adjuvant chemotherapy, while patients in the surgery alone (SA) group underwent surgery alone.

### Variables

The following preoperative clinical variables were evaluated: age, sex, ASA classification, BMI, pre-existing disease (e.g., hypertension, diabetes, heart disease, pulmonary disease), tumor sidedness, preoperative carcinoembryonic antigen(CEA) level and presence of perforation/obstruction. Additionally, the operation type, postoperative complications and pathologic variables (e.g., pT, harvested lymph nodes [LN] and lymphatic, venous and perineural invasion) were reviewed. Heart disease included ischemic heart disease (e.g., myocardial infarction, angina), arrhythmia, valvular disease and chronic heart failure. Pulmonary disease included chronic obstructive pulmonary disease (COPD), asthma and previous tuberculosis. Cancers from the cecum to transverse colon were defined as right-sided, while those from the splenic flexure to sigmoid colon were defined as left-sided. Complications was classified using the Clavien–Dindo classification. High-risk features included a poorly differentiated histology, perforation, bowel obstruction, <12 examined LN, lymphatic/vascular invasion or perineural invasion, according to the National Comprehensive Cancer Network guideline^13^.

### Procedure

All patients underwent curative resection, including D2 LN dissection. Adjuvant chemotherapy was administered 4 weeks postoperatively if the patient was deemed to have recovered. Most patients received the planned cycle of a fluorouracil (FU)-based chemotherapy regimen. All patients were recommended to attend follow-up visits every 3–6 months for the first 2 years and every 6 months thereafter for a total of 5 years. During these regular follow-ups, recurrences were detected through colonoscopy, computed tomography (CT) or magnetic resonance imaging (MRI) examinations.

### Survival data

The follow-up of older patients may be challenging. Therefore, survival data were obtained from Statistics Korea (KOSTAT), which records the date and cause of each death and is updated every 2 years. The most recent update occurred on December 31, 2016. The causes of death are stored using the International Classification of Diseases (ICD) code corresponding to the version current at the time of recording. Death from colon cancer was recorded as C18 during the period of 2002–2016.

### Primary outcomes

The primary endpoints were RFS, CSS and OS, which were compared between groups. RFS was calculated from the date of operation to the date of diagnosis of recurrence or death from any cause. CSS and OS were calculated from the date of operation to the date of death from colon cancer and to the date of death from any cause, respectively.

### Statistical analysis

SPSS version 25.0 for Windows (IBM Corp, Armonk, NY, USA) was used for the statistical analysis. A p value < 0.05 was considered statistically significant. Categorical baseline characteristics were analyzed using the χ^2^-test or linear-by-linear association, and continuous variables were analyzed using Student’s t-test. The two groups of patients were balanced using propensity score matching, which included a logistic regression with 1:1 nearest neighbor matching and a caliper of 0.2. The following covariables included: age, sex, ASA classification, BMI, perforation, obstruction, HTN, cardiac disease, pulmonary disease, tumor sidedness, operation type, tumor differentiation, size, pT, harvested LN, lymphatic invasion, venous invasion, perineural invasion

Kaplan–Meier curves and the log-rank test were used to evaluate the 5-year RFS, CSS and OS rates. A Cox regression hazard model was generated to identify the factors significantly affecting RFS, CSS and OS, and the multivariable analysis included factors with a p value < 0.2 in the univariable analysis.

## Results

**Figure 1:**
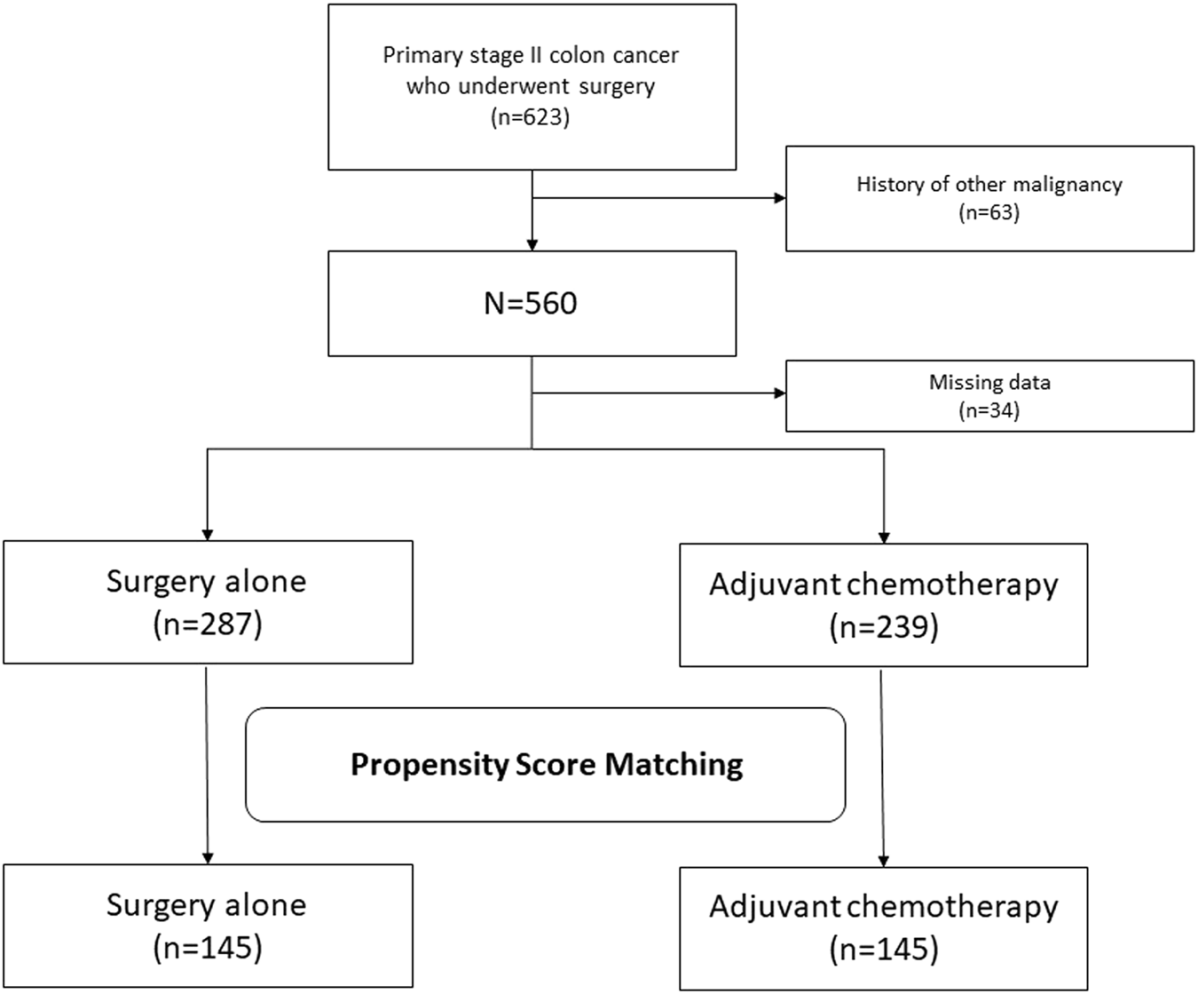
Flow chart of patient selection.

A total of 623 patients underwent curative resection at our institution between 2002 and 2015. Of these, 63 patients and 34 patients were excluded because of a history of other malignancy and missing data, respectively. Finally, 526 patients were included in our analysis (Fig. 1). Their baseline characteristics are presented in Table 1. Briefly, the overall mean age was 75.7 years (range: 70–93 years), and there was a slight male predominance (300/526, 57%).

**Table 1:** Baseline characteristics.

	Surgery alone (n = 287)	Adjuvant chemotherapy (n = 239)	p-value
Age (years)	77.8 ± 5.0	73.2 ± 2.8	0.01
Sex			0.514
Male	160 (55.7%)	140 (58.6%)	
Female	127 (44.3%)	99 (41.4%)	
BMI (kg/m^2^)	22.3 ± 3.3	23.1 ± 3.1	0.01
ASA classification			0.003
1	38 (13.2%)	55 (23.0%)	
2	214 (74.6%)	164 (68.6%)	
3	35 (12.2%)	20 (8.4%)	
Diabetes	64 (22.3%)	53 (22.2%)	0.973
Hypertension	168 (58.5%)	114 (47.7%)	0.013
Cardiac disease	27 (9.4%)	13 (5.4%)	0.087
Pulmonary disease	24 (8.4%)	17 (7.1%)	
Tumor side			0.529
Right-sided	129 (44.9%)	114 (47.7%)	
Left-sided	158 (55.1%)	125 (52.3%)	
Preoperative CEA (ng/mL)	8.9 ± 34.0	5.7 ± 10.6	0.146
Perforation	8 (2.8%)	5(2.1%)	0.609
Obstruction	89 (31.0%)	50 (20.9%)	0.009
Operation type			0.487
Open	227 (79.1%)	183 (76.6%)	
Laparoscopy	60 (20.9%)	56 (23.4%)	
Tumor differentiation			0.096
WD	13 (4.5%)	16 (6.7%)	
MD	245 (85.4%)	209 (87.4%)	
PD	15 (5.2%)	5 (2.1%)	
Mucinous	8 (2.8%)	7 (2.9%)	
Others	6 (2.1%)^a^	2 (0.8%)^b^	
Size (cm)	5.7 ± 2.4	5.3 ± 2.3	0.029
Pathologic T stage			0.113
3	259 (90.2%)	205 (85.8%)	
4	28 (9.8%)	34 (12.2%)	
The number of harvested LN			0.962
<12	44 (15.3%)	37 (15.5%)	
≥12	243 (84.7%)	202 (84.5%)	
Lymphatic invasion	47 (16.4%)	46 (19.2%)	0.39
Venous invasion	15 (5.2%)	25 (10.5%)	0.024
Perineural invasion	53 (18.5%)	59 (24.7%)	0.083
Postoperative complication	40 (13.9%)	25 (10.5%)	0.228
Clavien–Dindo classification			0.075
1	18	15	
2	6	2	
3	8	7	
4	7	1	
5	1	0	

^a^Medullary carcinoma (1), serrated adenocarcinoma (3), mixed adenoneuroendocrine carcinoma (1) and unknown information for differentiation (1).

^b^Serrated adenocarcinoma and adenosquamous carcinoma.

BMI, body mass index; ASA classification, American Society of Anesthesiologists physical status classification; CEA, carcinoembryonic antigen; WD, well-differentiated; MD, moderately differentiated; PD, poorly differentiated; LN, lymph node.

In an initial group comparison, patients in the SA group were older and had a lower BMI, higher ASA classification, larger tumor size and more frequent venous invasion, compared to the AC group. After propensity score matching to balance the pre-existing and pathologic variables, 145 patients were assigned to each arm. The mean ages of the matched SA and AC groups were 74.3 and 74.0 years, respectively (Table 2). In the AC group, after propensity score matching, the regimens followed were: 5-FU (n = 61), capecitabine (n = 40), capecitabine and oxaliplatin (n = 1), uracil/tegafur (UFT; n = 18), folinic acid-FU-oxaliplatin (FOLFOX; n = 21), and unknown (as the patients received chemotherapy at other hospitals; n = 4).

**Table 2:** Baseline characteristics after propensity score matching.

	Surgery alone (n = 145)	Adjuvant chemotherapy(n = 145)	p-value
Age (years)	74.3 ± 3.0	74.0 ± 3.0	0.309
Sex			0.473
Male	89 (61.4%)	83 (57.2%)	
Female	56 (38.6%)	62 (42.8%)	
BMI (kg/m^2^)	22.6 ± 3.4	22.8 ± 2.7	0.6
ASA classification			0.106
1	19 (13.1%)	34 (23.4%)	
2	109 (75.2%)	94 (64.8%)	
3	17 (11.7%)	17 (11.7%)	
Diabetes	35 (24.1%)	35 (24.1%)	>0.999
Hypertension	77 (53.1%)	76 (52.4%)	0.906
Cardiac disease	14 (9.7%)	11 (7.6%)	0.53
Pulmonary disease	13 (9.0%)	11 (7.6%)	0.67
Tumor side			0.813
Right-sided	64 (44.1%)	66 (45.5%)	
Left-sided	81 (55.9%)	79 (54.5%)	
Preoperative CEA (ng/mL)	9.7 ± 44.3	5.9 ± 11.8	0.341
Perforation	4 (2.8%)	2 (1.4%)	0.684
Obstruction	39 (26.9%)	35 (24.1%)	0.59
Operation type			0.666
Open	116 (80.0%)	113 (77.9%)	
Laparoscopy	29 (20.0%)	32 (22.1%)	
Tumor differentiation			0.434
WD	8 (5.5%)	8 (2.1%)	
MD	125 (86.2%)	128 (88.3%)	
PD	6 (4.1%)	3 (2.1%)	
Mucinous	3 (2.1%)	5 (3.4%)	
Others	3 (2.1%)^a^	1 (0.7%)^b^	
Size (cm)	5.3 ± 2.2	5.2 ± 2.2	0.69
Pathologic T stage			0.312
3	129 (89%)	134 (92.4%)	
4	16 (11.0%)	11 (7.6%)	
The number of harvested LN			0.441
<12	28 (19.3%)	23 (15.9%)	
≥12	117 (80.7%)	122 (84.1%)	
Lymphatic invasion	23 (15.9%)	18 (12.4%)	0.399
Venous invasion	6 (4.8%)	6 (4.8%)	>0.999
Perineural invasion	32 (22.1%)	35 (24.1%)	0.676
Postoperative complication	17 (11.7%)	15 (10.3%)	0.708
Clavien-Dindo classification			0.149
1	6	10	
2	4	2	
3	2	2	
4	4	1	
5	1	0	

^a^Medullary carcinoma (1) and serrated adenocarcinoma (2).

^b^Adenosquamous carcinoma.

BMI, body mass index; ASA classification, American Society of Anesthesiologists physical status classification; CEA, carcinoembryonic antigen; WD, well-differentiated; MD, moderately differentiated; PD, poorly differentiated; LN, lymph node.

### Recurrence-free survival (RFS)

**Figure 2:**
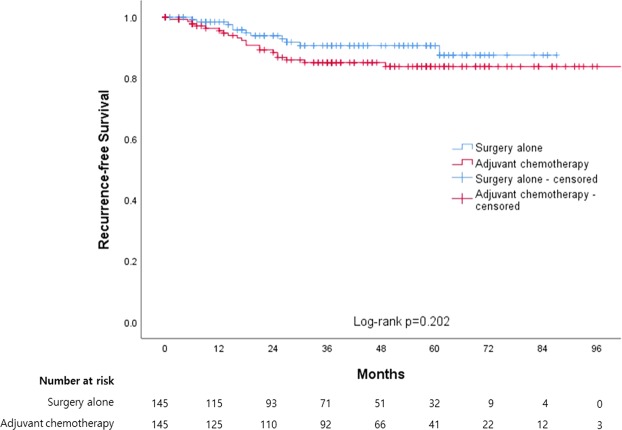
Kaplan-Meier curves between surgery alone and adjuvant chemotherapy for recurrence-free survival.

All patients were followed to detect recurrence for a mean of 1337.7 days (range: 15–3403 days). Recurrence was detected in 11 (7.6%) and 20 patients (13.8%) in the SA and AC groups, respectively, which had median RFS durations of 79.8 (95% confidence interval [CI]: 75.8–83.9) and 96.1 months (95% CI: 90.1–102.1), respectively, as determined using Kaplan–Meier curves (Fig. 2). The corresponding 5-year RFS rates were 91.8 and 85.1%, respectively, and this difference was not statistically significant (log-rank test, p = 0.202).

### Cancer-specific survival and overall survival

**Figure 3:**
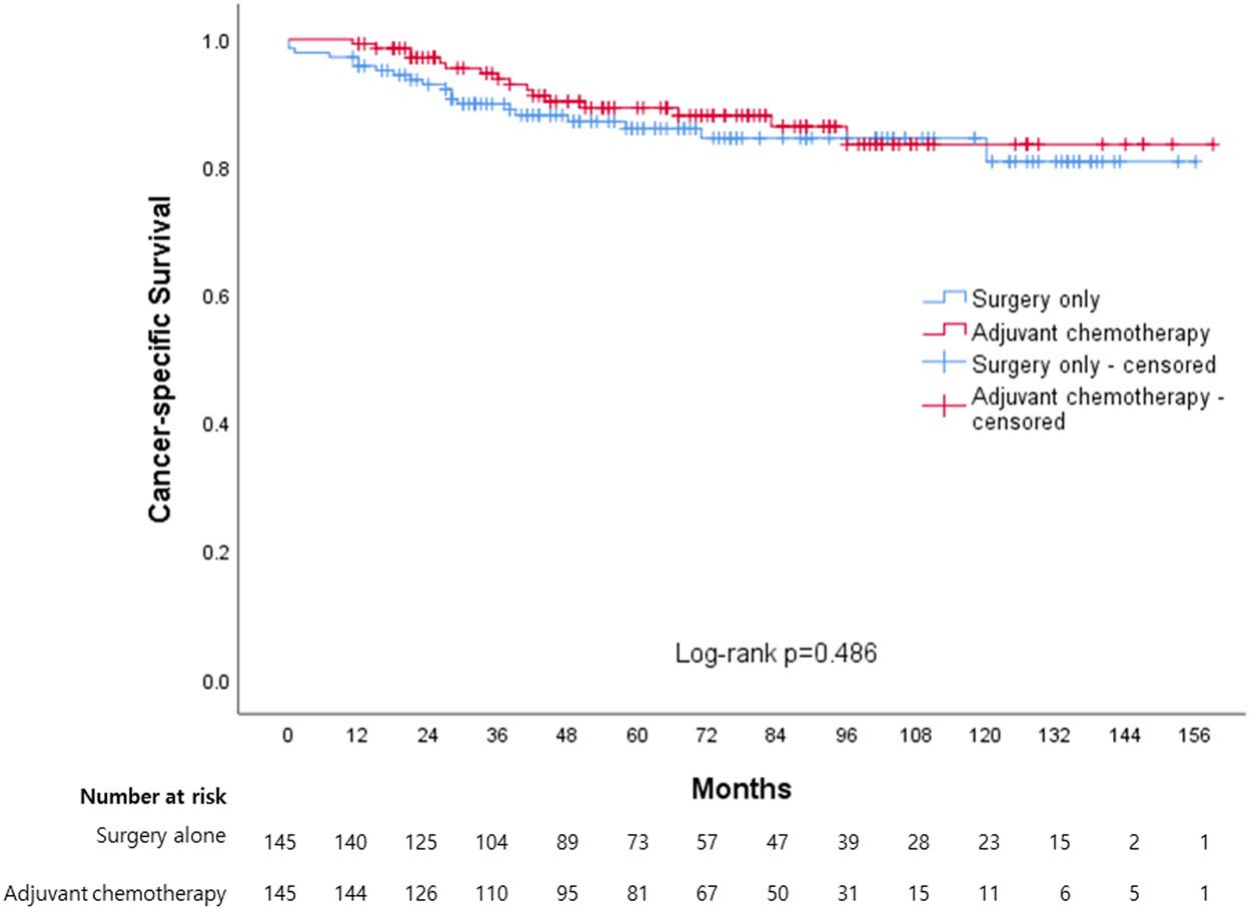
Kaplan-Meier curves between surgery alone and adjuvant chemotherapy for cancer-specific survival.

The mean survival follow-up duration was 2049.5 days (range: 26–4853 days). Thirty-eight (26.2%) and 29 patients (20.0%) in the SA and AC groups, respectively, died during this period, and the cause of death was colon cancer in 20 and 16 patients, respectively. A Kaplan–Meier analysis yielded median CSS durations of 135.0 (95% CI: 126.6–143.5) and 141.3 months (95% CI: 133.1–149.5) months in the SA and AC groups, respectively, which had 5-year CSS rates of 86.0 and 89.3%, respectively (Fig. 3). This difference was not statistically significant (log-rank test, p = 0.486).

**Figure 4:**
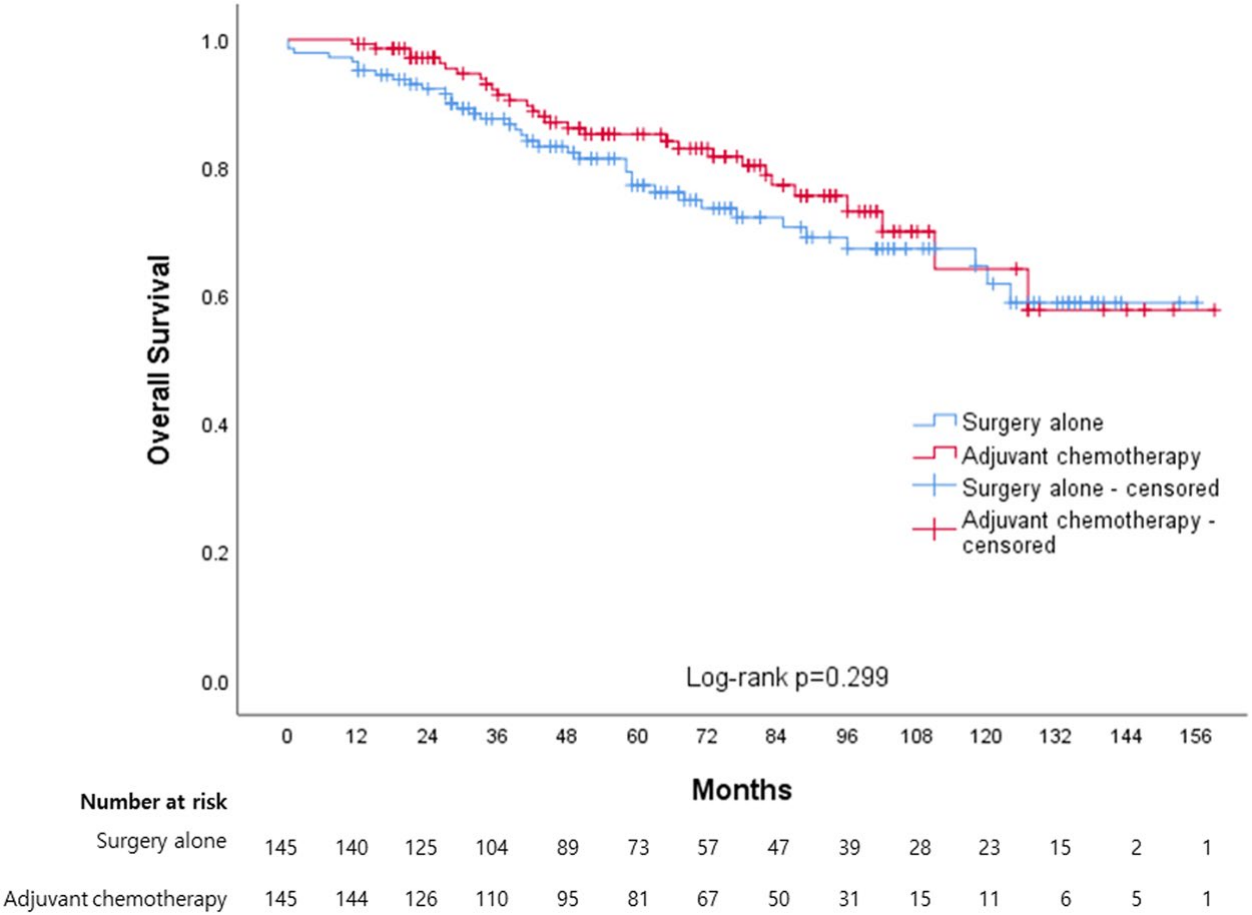
Kaplan-Meier curves between surgery alone and adjuvant chemotherapy for overall survival.

The median OS durations in the SA and AC groups were 117.3 (95% CI: 107.2–127.4) and 124.5 months (95% CI: 113.4–135.5) months, respectively (Fig. 4). The corresponding 5-year OS rates were 81.4 and 85.2%, and this difference was not statistically significant (log-rank test, p = 0.299).

### Factors associated with RFS, CSS and OS

#### RFS

In a univariable analysis, age, pulmonary disease, preoperative CEA level and lymphatic, venous and perineural invasion were identified as statistically significant factors for RFS. In a multivariable analysis, the CEA level (hazard ratio [HR]: 1.01, 95% CI: 1.00–1.01), venous invasion (HR: 3.57, 95% CI: 1.15–11.06) and perineural invasion (HR: 3.40, 95% CI: 1.47–7.85) remained independent and significant factors affecting RFS (Table 3). However, adjuvant chemotherapy was not identified as a significant factor (HR: 1.61, 95% CI: 0.77–3.36).

**Table 3:** Univariable and multivariable analyses to identify factors affecting recurrence-free survival, cancer specific survival and overall survival.

	RFS				CSS				OS			
	Univariable		Multivariable^†^		Univariable		Multivariable^†^		Univariable		Multivariable^†^	
	HR (95% CI)	*P*	HR (95% CI)	*P*	HR (95% CI)	*P*	HR (95% CI)	*P*	HR (95% CI)	*P*	HR (95% CI)	*P*
Age (years)	1.12 (1.00–1.25)	0.044	1.01 (0.89–1.15)	0.9	1.15 (1.03–1.28)	0.01	1.05 (0.92–1.21)	0.437	1.11 (1.02–1.21)	0.016	1.07 (0.98–1.17)	0.158
Sex		0.195		0.116		0.192		0.126		0.003		0.003
Male	Reference		Reference		Reference		Reference		Reference		Reference	
Female	0.61 (0.29–1.29)		0.49 (0.20–1.19)		0.62 (0.31–1.27)		0.53 (0.24–1.19)		0.42 (0.24–0.74)		0.40 (0.21–0.73)	
BMI (kg/m^2^)	1.03 (0.92–1.16)	0.615			0.879 (0.78–0.99)	0.03	0.97 (0.84–1.11)	0.65	0.89 (0.82–0.97)	0.009	0.93 (0.84–1.03)	0.171
ASA		0.35				0.056		0.13		0.037		0.08
1	Reference				Reference		Reference		Reference		Reference	
≥2	1.65 (0.58–4.72)				4.01 (0.96–16.70)		4.82 (0.63–36.89)		2.45 (1.06–5.66)		2.58 (0.89–7.43)	
Diabetes	1.26 (0.56–2.82)	0.575			1.41 (0.69–2.87)	0.342			0.92 (0.52–1.64)	0.781		
Hypertension	1.39 (0.68–2.84)	0.364			1.22 (0.63–2.36)	0.558			1.52 (0.93–2.49)	0.094	1.66 (0.95–2.91)	0.075
Cardiac disease	1.53 (0.54–4.39)	0.424			1.07 (0.33–3.49)	0.91			1.60 (0.76–3.35)	0.214		
Pulmonary disease	2.94 (1.13–7.67)	0.027	1.00 (0.26–3.85)	0.998	2.51 (0.97–6.48)	0.057	1.73 (0.44–6.82)	0.437	2.14 (1.02–4.49)	0.045	3.02 (1.20–7.59)	0.018
Tumor side		0.531				0.223				0.291		
Right-sided	Reference				Reference				Reference			
Left-sided	1.26 (0.62–2.57)				1.53 (0.77–3.02)				1.30 (0.80–2.12)			
CEA (ng/mL)	1.01 (1.01–1.01)	<0.001	1.01 (1.00–1.01)	0.004	1.01 (1.01–1.01)	<0.001	1.01 (1.00–1.01)	0.005	1.01 (1.01–1.01)	<0.001	1.01 (1.00–1.01)	<0.001
Perforation	2.09 (0.28–15.35)	0.469			3.70 (0.89–15.45)	0.073	1.49 (0.11–19.56)	0.763	4.74 (1.71–13.09)	0.003	0.88 (0.09–9.05)	0.916
Obstruction	1.84 (0.88–3.85)	0.104	1.42 (0.60–3.38)	0.425	3.22 (1.67–6.19)	<0.001	2.95 (1.33–6.56)	0.763	1.98 (1.21–3.24)	0.007	1.79 (1.03–3.10)	0.039
Operation type		0.094		0.167		0.865				0.199		0.292
Open	Reference		Reference		Reference				Reference		Reference	
Laparoscopy	1.91 (0.90–4.05)		1.88 (0.77–4.62)		0.93 (0.39–2.23)				0.60 (0.27–1.31)		0.61 (0.24–1.54)	
Size (cm)	1.05 (0.89–1.23)	0.603			1.20 (1.05–1.38)	0.008	1.19 (1.02–1.40)	0.025	1.14 (1.03–1.27)	0.014	1.21 (1.07–1.37)	0.003
Pathologic T stage		0.304				<0.001		0.377		0.002		0.637
3	Reference				Reference		Reference		Reference		Reference	
4	1.74 (0.61–4.97)				4.78 (2.23–10.22)		1.37 (0.46–4.06)		2.92 (1.48–5.74)		1.24 (0.51–2.99)	
Harvested LNs		0.743				0.434				0.087		0.002
<12	Reference				Reference				Reference		Reference	
≥12	0.86 (0.35–2.10)				0.74 (0.35–1.58)				0.63 (0.37–1.07)		0.37 (0.19–0.70)	
Lymphatic invasion	2.78 (1.28–6.04)	0.01	2.03 (0.79–5.26)	0.143	1.87 (0.85–4.10)	0.12	0.82 (0.30–2.22)	0.692	1.87 (1.05–3.32)	0.034	1.30 (0.66–2.56)	0.454
Venous invasion	4.88(1.87–12.76)	0.001	3.57 (1.15–11.06)	0.028	6.70 (2.75–16.30)	<0.001	5.55 (2.04–15.06)	0.001	3.51 (1.50–8.19)	0.004	3.41 (1.38–8.42)	0.008
Perineural invasion	4.05 (1.99–8.26)	<0.001	3.40 (1.47–7.85)	0.004	3.75(1.91–7.34)	<0.001	2.34 (1.03–5.29)	0.041	1.89 (1.07–3.34)	0.028	1.33 (0.67–2.64)	0.407
Postoperative complication	2.37 (0.97–5.79)	0.057	2.03 (0.68–6.08)	0.204	2.56 (1.11–5.90)	0.027	2.02 (0.72–5.69)	0.182	2.61 (1.35–5.05)	0.004	1.56 (0.72–3.38)	0.26
Adjuvant chemotherapy	1.61 (0.77–3.36)	0.206			0.79 (0.41–1.53)	0.487			0.77 (0.48–1.26)	0.301		

^†^Multivariable analysis included factors with p-values < 0.20 in the univariable analysis.

HR, hazard ratio; 95% CI, 95% confidence interval; BMI, body mass index; ASA, American Society of Anesthesiologists physical status classification; CEA, preoperative carcinoembryonic antigen level; LN, lymph node.

#### CSS

In a univariable analysis, age, BMI, obstruction, preoperative CEA level, tumor size, pathologic T stage, venous and perineural invasion and postoperative complications were identified as statistically significant factors affecting CSS. In a multivariable analysis, CEA (HR: 1.01, 95% CI: 1.00–1.01), tumor size (HR: 1.19, 95% CI: 1.02–1.40) and venous (HR: 5.55, 95% CI: 2.04–15.06) and perineural invasion (HR: 2.34, 95% CI: 1.03–5.29) remained independent and significant factors affecting CSS (Table 3). Again, however, adjuvant chemotherapy was not statistically significant (HR: 0.79, 95% CI: 0.41–1.53).

#### OS

In a univariable analysis, age, sex, BMI, ASA, pulmonary disease, perforation, obstruction, preoperative CEA level, tumor size, pathologic T stage, postoperative complications and lymphatic, venous and perineural invasion were identified as statistically significant. In a multivariable analysis, female sex (HR: 0.40, 95% CI: 0.21–0.73), pulmonary disease (HR: 3.02, 95% CI: 1.20–7.59), CEA (HR: 1.01, 95% CI 1.00–1.01), obstruction (HR: 1.79, 95% CI: 1.03–3.10), size (HR: 1.21, 95% CI: 1.07–1.37), harvested LN ( > 12) (HR: 0.37, 95% CI: 0.19–0.70), and venous invasion (HR: 3.41, 95% CI: 1.38–8.42) were independent factors affecting OS (Table 3). However, adjuvant chemotherapy was not a statistically significant factor (HR: 0.77, 95% CI: 0.48–1.26).

### Subgroup analysis

We further divided patients into low- and high-risk subgroups to analyze the effect of adjuvant chemotherapy. The high-risk subgroup was defined as patients who fulfilled one or more of the following criteria: poorly differentiated histology, perforation, bowel obstruction, < 12 examined LNs, lymphatic/vascular invasion, and perineural invasion. However, this factor did not affect RFS, CSS, or OS in either the low- or high-risk patient subgroups. A multivariable analysis revealed no significant factors (Table 4). Finally, we analyzed the factors affecting RFS, CSS and OS in the AC group. Notably, perineural invasion was an independent factor affecting RFS (HR: 5.03, 95% 1.89–13.40) and CSS (HR: 3.99, 95% CI 1.24–12.84), while obstruction and postoperative complications were associated with CSS (HR: 4.16, 95% CI: 1.22–14.14 and HR: 5.24, 95% CI: 1.33–20.61, respectively) and OS (HR: 2.74, 95% CI: 1.14–6.62 and HR: 3.35, 95% CI: 1.10–10.19, respectively) (Table 5).

**Table 4:** Effects of adjuvant chemotherapy on recurrence-free survival, cancer-specific survival and overall survival in patients with stage II colon cancer with low-risk and high-risk features.

	Unadjusted HR (95% CI)	p-value	Adjusted HR^a^ (95% CI)	p-value
**Recurrence-free survival**				
Low risk		0.921		0.497
Surgery alone	Reference		Reference	
Adjuvant chemotherapy	0.93 (0.23–3.74)		0.50 (0.07–3.63)	
High risk		0.165		0.143
Surgery alone	Reference		Reference	
Adjuvant chemotherapy	1.88 (0.77–4.56)		2.19 (0.77–6.25)	
**Cancer-specific survival**				
Low risk		0.095		0.528
Surgery alone	Reference		Reference	
Adjuvant chemotherapy	0.38 (0.12–1.18)		0.66 (0.18–2.44)	
High risk		0.608		0.942
Surgery alone	Reference		Reference	
Adjuvant chemotherapy	1.25 (0.53–2.98)		1.04 (0.35–3.07)	
**Overall survival**				
Low risk		0.054		0.364
Surgery alone	Reference		Reference	
Adjuvant chemotherapy	0.49 (0.24–1.01)		0.70 (0.32–1.52)	
High risk		0.722		0.649
Surgery alone	Reference		Reference	
Adjuvant chemotherapy	1.13 (0.57–2.25)		0.83 (0.36–1.89)	

^a^Adjusted factors: age, sex, American Society of Anesthesiologists classification, body mass index, diabetes, hypertension, cardiac disease, pulmonary disease, tumor side, preoperative carcinoembryonic antigen level, operation type, tumor size, pathologic T stage and postoperative complication

FL, 5-fluorouracil, leucovorin; HR, hazard ratio; CI, confidence interval.

**Table 5:** Subgroup analysis for recurrence-free survival, cancer-specific survival and overall survival in adjuvant chemotherapy group.

	RFS				CSS				OS			
	Univariable		Multivariable^†^		Univariable		Multivariable^†^		Univariable		Multivariable^†^	
	HR (95% CI)	*P*	HR (95% CI)	*P*	HR (95% CI)	*P*	HR (95% CI)	*P*	HR (95% CI)	*P*	HR (95% CI)	*P*
Age (years)	1.13 (0.99–1.29)	0.067	1.06 (0.90–1.24)	0.496	1.21 (1.04–1.40)	0.014	1.14 (0.96–1.35)	0.124	1.16 (1.03–1.31)	0.015	1.12 (0.97–1.28)	0.127
Sex		0.689				0.242				0.018		0.071
Male	Reference				Reference				Reference		Reference	
Female	0.83 (0.34–2.04)				0.51 (0.16–1.58)				0.31 (0.12–0.82)		0.39 (0.14–1.08)	
BMI (kg/m^2^)	1.09 (0.93–1.27)	0.27			0.92 (0.75–1.13)	0.42			0.97 (0.83–1.12)	0.652		
ASA		0.348				0.324				0.119		0.228
1	Reference				Reference				Reference		Reference	
≥2	1.80 (0.53–6.15)				2.11 (0.48–9.28)				2.59 (0.78–8.56)		2.49 (0.57–10.93)	
Diabetes	1.17 (0.43–3.23)	0.757			0.65 (0.19–2.29)	0.504			0.69 (0.28–1.69)	0.41		
Hypertension	1.53 (0.62–3.74)	0.354			1.17 (0.43–3.17)	0.757			1.64 (0.77–3.51)	0.204		
Cardiac disease	0.64 (0.09–4.76)	0.66			0.05 (0.00–310.55)	0.492			1.24 (0.29–5.25)	0.769		
Pulmonary disease	0.66 (0.09–4.91)	0.683			1.11 (0.15–8.39)	0.922			2.41 (0.83–6.99)	0.105	5.28 (1.68–16.56)	0.004
Tumor sideness		0.889				0.514				0.518		
Right sided	Reference				Reference				Reference			
Left sided	1.07 (0.44–2.57)				1.40 (0.51–3.86)				1.28 (0.61–2.70)			
CEA (ng/mL)	1.03 (1.01–1.05)	0.014	1.01 (0.99–1.04)	0.249	1.04 (1.02–1.06)	<0.001	1.03 (1.01–1.05)	0.011	1.03 (1.02–1.05)	<0.001	1.02 (1.00–1.04)	0.092
Perforation	4.91 (0.66–36.78)	0.122	5.59 (0.29–108.74)	0.255	6.53 (0.84–50.82)	0.073	5.97 (0.12–286.87)	0.366	4.41 (0.58–33.36)	0.151	1.83 (0.13–25.36)	0.653
Obstruction	1.52 (0.58–3.96)	0.391			3.38 (1.27–9.04)	0.015	4.16 (1.22–14.14)	0.022	2.29 (1.09–4.80)	0.029	2.74 (1.14–6.62)	0.025
Operation type		0.77				0.277				0.236		
Open	Reference				Reference				Reference			
Laparoscopy	1.16 (0.42–3.21)				0.33 (0.04–2.46))				0.42 (0.10–1.77)			
Size (cm)	1.09 (0.89–1.32)	0.413			1.19 (0.96–1.48)	0.113	0.94 (0.65–1.36)	0.741	1.06 (0.89–1.25)	0.514		
Pathologic T stage												
3	Reference	0.763			Reference	0.23			Reference	0.536		
4	1.25 (0.29–5.40)				2.49 (0.56–11.02)				1.58 (0.37–6.71)			
Harvested LNs												
<12	Reference	0.296			Reference	0.141	Reference	0.4	Reference	0.006		
≥12	0.58 (0.21–1.60)				0.45 (0.16–1.30)		0.53 (0.12–2.35)		0.35 (0.16–0.74)			
Lymphatic invasion	2.47 (0.90–6.80)	0.08	1.65 (0.47–5.77)	0.43	3.13 (1.00–9.75)	0.049	1.74 (0.35–8.63)	0.499	3.04 (1.29–7.18)	0.011	0.50 (0.19–1.31)	0.157
Venous invasion	2.27 (0.53–9.79)	0.271			3.64 (0.82–16.11)	0.089	2.57 (0.37–17.99)	0.341	1.49 (0.34–6.47)	0.595		
Perineural invasion	4.45 (1.83–10.81)	0.001	5.03 (1.89–13.40)	0.001	4.24 (1.49–12.13)	0.007	3.99 (1.24–12.84)	0.02	2.72 (1.13–6.66)	0.025	0.50 (0.20–1.26)	0.142
Postoperative complication	2.57 (0.86–7.68)	0.092			3.78 (1.21–11.85)	0.023	5.24 (1.33–20.61)	0.018	2.85 (1.07–7.61)	0.036	3.35 (1.10–10.19)	0.033

^†^Multivariable analysis included factors with p-values < 0.20 in the univariable analysis.

HR, Hazard ratio; 95% CI, 95% confidence interval; BMI, body mass index; ASA, The American Society of Anesthesiologists (ASA) physical status classification; CEA, Preoperative Carcinoembryonic Antigen level; LN, lymph node.

To evaluate the effect of adding oxaliplatin to 5-FU in elderly patients, we divided the patients who received chemotherapy into two groups: those who received 5-FU (n = 119) and those who received 5-FU and oxaliplatin (n = 22). There was no statistically significant difference in the 5-year RFS (86.5% vs. 70.2%, log-rank p = 0.111), CSS (90.2% vs. 87.9%, log-rank p = 0.743), or OS (86.3% vs. 87.9%, log-rank p = 0.816) between the two groups. Moreover, adding oxaliplatin did not significantly affect the RFS (HR 2.24, 95% CI 0.81–6.62), CSS (HR 0.84, 95% CI 0.20–3.60), or OS (HR 0.84, 95% CI 0.20–3.60).

## Discussion

Our study shows that postoperative adjuvant chemotherapy confers no major survival benefit in patients older than 70 years with stage II colon cancer, even after propensity score matching. Additionally, adjuvant chemotherapy did not affect the RFS, CSS, or OS outcomes even in patients with high-risk features. These findings may have a positive impact on many patients, especially those at an advanced age, who can avoid the problems associated with chemotherapy.

As noted previously, no consensus has been reached regarding the effect of adjuvant chemotherapy for stage II colon cancer. The QUASAR trial reported improved survival outcomes in patients with stage II colon cancer who received chemotherapy comprising FU and folinic acid^9^, while Casadaban *et al*. reported better OS in patients with stage II colon cancer included in a national cancer database^8^. However, recent studies including elderly patients, in contrast to the QUASAR trial and the study by Casadaban *et al*., have failed to demonstrate an effect of adjuvant chemotherapy. For example, Booth *et al*. reported no benefit of adjuvant chemotherapy in terms of CSS and OS even in high-risk patients in a population-based study^14^, consistent with our findings. These results suggest that management guidelines for stage II patients should be redefined.

There is some debate about the role of chemotherapy in elderly patients with colorectal cancer. The Adjuvant Colon Cancer End Points Collaborative Group showed that elderly patients (aged ≥70 years) with stage II/III colon cancer did not experience a statistically significant benefit from adjuvant chemotherapy in terms of disease-free survival (HR, 1.05; 95% CI, 0.94 to 1.19) and OS (HR, 1.08; 95% CI, 0.95 to 1.23)^15^. Popescu *et al*., in their study of first-line chemotherapy for patients with advanced colorectal cancer in elderly patients (≥70), reported that the median OS period was shorter in the elderly group than in younger patients (292 vs. 350 days, p = 0.04)^16^. Strowitzki *et al*. showed that even in a total of 468 patients with colorectal liver metastases the administration of neoadjuvant chemotherapy is of questionable value^17^.

However, there are authors who have shown positive effects of chemotherapy on elderly patients. Fata *et al*. reported that elderly patients with stage II/III colon cancer benefit from 5-FU-based adjuvant therapy, without a significant increase in toxicity compared to that in their younger counterparts^18^. There is even an article about the benefit of adjuvant therapy after curative resection in stage IV colon cancer. Rahbari *et al*. analysed a total of 297 patients with curative resection of colorectal cancer liver metastasis. According to their results, adjuvant chemotherapy was associated with improved survival in the entire cohort (HR 0.69; 95% CI 0.69–0.98)^19^.

Some studies have evaluated the efficacy of adjuvant chemotherapy, particularly in patients older than 70 years with stage II colon cancer. For example, Tsai *et al*. reported no significant difference in OS between patients who received adjuvant chemotherapy and those without adjuvant chemotherapy^6^. However, that study compared two groups, adjuvant chemotherapy vs. no adjuvant chemotherapy, that were unbalanced in terms of baseline characteristics such that the former group had a worse pathologic grade and larger proportion of pT4 stage disease. In a large Korean database study^20^, Kim *et al*. performed a subgroup analysis of patients older than 70 years with stage II colon cancer and concluded that adjuvant chemotherapy yielded an OS benefit. However, Kim and colleagues also reported that their two groups were unbalanced in terms of baseline characteristics, and limitations of their dataset precluded analyses of RFS and CSS. By contrast, we performed propensity score matching to balance the study groups and conducted analyses of both RFS and CSS. These represent strengths of our study.

In our multivariable analysis of patients who received adjuvant chemotherapy, we identified perineural invasion, a well-known prognostic factor for stage II colon cancer^13^, as a statistically independent factor affecting RFS and CSS. Furthermore, CEA and tumour size also affected CSS and OS in multivariable analysis. A higher pretreatment CEA level has been identified as a predictor of RFS and OS^21–23^. In particular, an elevated CEA level preoperatively in early stage colon cancer has been associated with a poor prognosis compared with normal CEA levels in a node-positive tumour^24^. Tumour size is also a factor associated with a poor oncologic prognosis^25,26^.

We further identified obstruction and postoperative complications as significant factors affecting CSS and OS and confirmed a lack of covariance between these factors (χ^2^-test, p > 0.999. We note that patients with obstruction had a lower BMI, compared to those without obstruction (21.6 vs. 23.1 kg/m^2^, p = 0.004), which indicates the need for careful attention to avoid postoperative complications during chemotherapy in fragile patients. Furthermore, a lower BMI is always associated with a worse condition. We recommend further study of cancer-related mortality after chemotherapy.

To our best knowledge, our study is first study of its type to apply propensity score matching to balance patient groups even with respect to co-morbidity. Our findings were consistent with other recent studies that found no difference in the oncologic outcomes of patients with stage II colon cancer who did and did not receive adjuvant chemotherapy. Taken together, these findings underscore the need to discuss guidelines for the management of geriatric patients with stage II colon cancer.

### Limitations

This study had some potential limitations. First, the retrospective design might have led to selection bias. Second, we were unable to obtain information about patients who received chemotherapy at a reduced dose or cycle number. Third, we included a relatively small number of patients, compared to other studies based on national databases. However, the total number of initially included patients was not small, and the 290 patients remaining after propensity score matching was sufficient for a statistical analysis. Fourth, we lacked data regarding why chemotherapy was not given to some patients, even in the high-risk subgroup. Although we were limited by an inability to confirm the Eastern Cooperative Oncology Group performance status, we attempted to eliminate differences in confirmed comorbidities between the two groups as much as possible. Fifth, in the medical records, there was no information about resection margin status, such as intermediate or close margin, according to the National Comprehensive Cancer Network guideline^13^, although information about other high-risk features was present. Our study only included patients who underwent curative resection (R0). For this reason, there were no patients with a positive margin.

## Conclusion

Adjuvant chemotherapy did not appear to confer RFS, CSS, or OS benefits in patients older than 70 years with stage II colon cancer. However, our finding that obstructive colon cancer and postoperative complications were associated with poorer survival outcomes suggests that patients meeting these criteria should be followed cautiously during chemotherapy. Our findings underscore the need to revise guidelines for the treatment of stage II colon cancer. As our study population is not representative of all patients with stage II disease, we believe that a well-balanced, large population-based study is warranted.
